# Phenotypic variation in 1,100 provenances of *Picea abies* measured over 50 years on 33 German trial sites

**DOI:** 10.1038/s41597-024-03726-x

**Published:** 2024-08-09

**Authors:** Katharina J. Liepe, Christoph A. Rieckmann, Hannah S. Mittelberg, Mirko Liesebach

**Affiliations:** 1 Thuenen Institute of Forest Genetics, Grosshansdorf, Germany; 2 Chair of Forest Growth and Woody Biomass Production, Technische Universität Dresden, Tharandt, Germany

**Keywords:** Forestry, Plant breeding

## Abstract

We present a database of Norway spruce phenotypic traits measured over a period of 51 years in provenance trials planted across western Germany. These trials are part of the ‘Inventory Provenance Test 1964/68 with Norway spruce’ (IPTNS) that tested a total of 1,100 provenances collected across Europe, both from the species natural and artificial range. Phenotypic traits (first height, later diameter at breast height, survival) were recorded at 33 trial sites of 100 provenances, each a subsample from the entire collection area that represents an approximately comparable climate space. The dataset contains 424,781 records of the same 65,518 trees ranging from age 7 to 51. Overall, it captures the considerable genetic and phenotypic intraspecific variation present in Norway spruce and should be of interest to various fields including quantitative genetics, ecology, biogeography and the adaptive management of forests.

## Background & Summary

Norway spruce (*Picea abies* (L.) Karst.) is suffering severely under the recent climate changes, leading to intensive discussions of the species role in future silviculture. Negative effects of climate warming in general are accelerated by extreme events, including pronounced drought and heat, storms and associated bark beetle infestations^1^. Species distribution models predict large scale range reductions^2,3^, first back towards the natural range, but also further within. However, Norway spruce is still considered one of the key tree species for European forestry, accounting for 23% of the total growing stock in 2020^4^. From an economic perspective the species appears almost indispensable due to its economic versatility and high ecological plasticity^5^. In addition, a dense natural regeneration is emerging in various previously disturbed stands, raising the question whether and how to integrate Norway spruce in the diverse forest structures anticipated for the future.

Scientific evidence of the extent of local adaptation of tree populations and their ability to acclimatize via phenotypic plasticity is urgently needed to develop management recommendations. This is important at the broad distributional level, but even more so at the local level, where recommendations need to be tailored for forest practitioners out in the field. In this regard, large and systematic provenance trials, testing populations collected throughout a range of source climates and grown together across a range of recipient climates, provide an essential data resource. These trials, originally planted to maximize the productivity of artificially regenerated forests with the best suited reproductive material^6^, have emerged as useful climate change laboratories^7^. Today they are used to predict the future potential of tree populations under rapidly changing climate. Reanalysing phenotypic trait expression assessed in these trials with state-of-the-art statistical approaches^8^, e.g., relating it to climate via response functions^9^ or universal response functions^3^, aids in predicting future growth performance, and in consequence climatic species suitability.

**Fig. 1:**
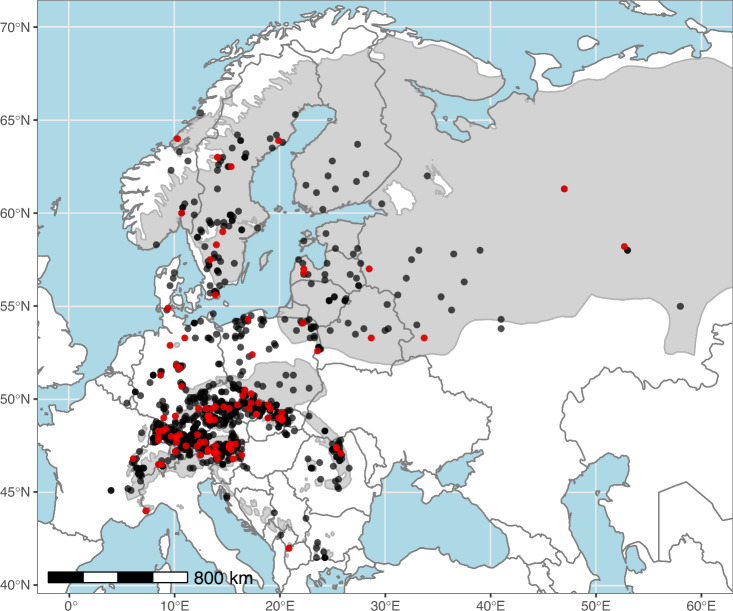
Geographic location of 1,100 tested provenances. To illustrate the stratified assignment of 100 provenances to each block (1 to 11), provenances of block 1 are highlighted in red. Provenances sourced east of the Ural Mountains are not shown in the given map extent. The natural distribution of *Picea abies* in grey in the background is reproduced from EUFORGEN^16^.

The largest provenance trial series by far, both in number of seed sources tested as well as number of countries participating in trial site establishment, was the ‘Inventory Provenance Test 1964/68 with Norway spruce’ (IPTNS) initiated by Olof Langlet (Royal College of Forestry, Stockholm, Sweden). Langlet intended to create an inventory to test ‘as many provenances as possible, regardless whether the seed sources were autochthonous or not, and regardless whether the sampled seed stands were located within or outside the natural distribution of Norway spruce’^10^. The trial series obtained IUFRO status at the 1967 Provenance Meeting of the International Union of Forest Research Organizations at Pont à Mousson, after having been a rather private enterprise. Thereafter, the trial series was also labelled ‘IUFRO 1964/68 provenance trial of Norway spruce’^10^. A total of 20 experiments, including 1,100 seed sources, were conducted by 13 participating countries in 1968 and 1969. Due to the large number of populations, the complete set of 1,100 seed sources was split into 11 blocks of 100 populations each (Fig. 1). These blocks were assembled based on a stratified sampling to contain material from the entire sampled area, approximately representing a comparable climate space.

Here, we present height and diameter data from three German experiments from this series (in total 33 trial sites with 100 provenances each) that were assessed for an observation period of 51 years. All sites were located in the species artificial range in western Germany, representing areas where Norway spruce was planted at high frequency in past afforestations. In combination with previously published data of this series^6,11–13^, these data can help to understand the entire gradient of climatic tolerance of Norway spruce. It may be used to investigate provenance differentiation at spatial scales and to determine their ability of phenotypically plastic response. Overall, the publication of data from such long-term experiments provides an invaluable resource helping us understand the capacity of forests to adapt and acclimatise to climate change. This data provides the urgently required, practically-relevant evidence to develop recommendations, but also to issue warnings, as to which the species in general and the various provenances will be suitable in the future^3,8^.

## Methods

### Provenance selection and nursery procedures

Seed sources of *Picea abies*, hereafter referred to as provenances, were selected using a sampling strategy developed by Peter Krutzsch (Royal College of Forestry, Stockholm, Sweden), who defined 95 focus regions, across the whole area of species occurrence. Seeds were collected over a period of four consecutive years, yielding a total of 1,615 seedlots in 1964. Due to geographic heterogeneity and the associated climatic differences, the selection density was particularly high in Central Europe and the Alpine distribution of the species (Fig. 1).

In spring 1964 1,300 seedlots were sown by the Institute of Forest Genetics and Tree Breeding of the Federal Research Centre for Forestry and Forest Products (now the Thuenen Institute of Forest Genetics, Grosshansdorf, Germany). After two years the seedlings were transplanted to Pein & Pein nursery (Halstenbeck, Germany) for another two years. In 1968 seedlings from 1,100 provenances were lifted, labelled, assorted and shipped to cooperating institutions in 13 countries (including Canada) for the establishment of 20 experiments with equal provenance representation. Detailed information about these 1,100 provenances, including seed source categories, focal regions of seed collection and derived provenance groupings, are provided by Ujvári-Jármay *et al*.^6^.

### Experimental design

The experimental design of the field trials was developed by Peter Krutzsch, in cooperation with Klaus Stern and Wolfgang Langner (Institute of Forest Genetics and Tree Breeding). The complete set of 1,100 provenances was split into 11 blocks of 100 unique populations each, based on a stratified sampling to contain material from the entire sampling area. These blocks can be regarded as a complete trial by themselves and were assumed to be equal in mean and within-block variance, thus easily comparable^14^. With exception of two experiments (in Scotland and Finland), all trial sites were established in a randomized complete block design with single-tree-plots of 20 or 25 replications to achieve sufficient experimental accuracy dealing with the large number of provenances^10^. The spacing between adjacent trees was predefined at 2 × 2 m.

**Fig. 2:**
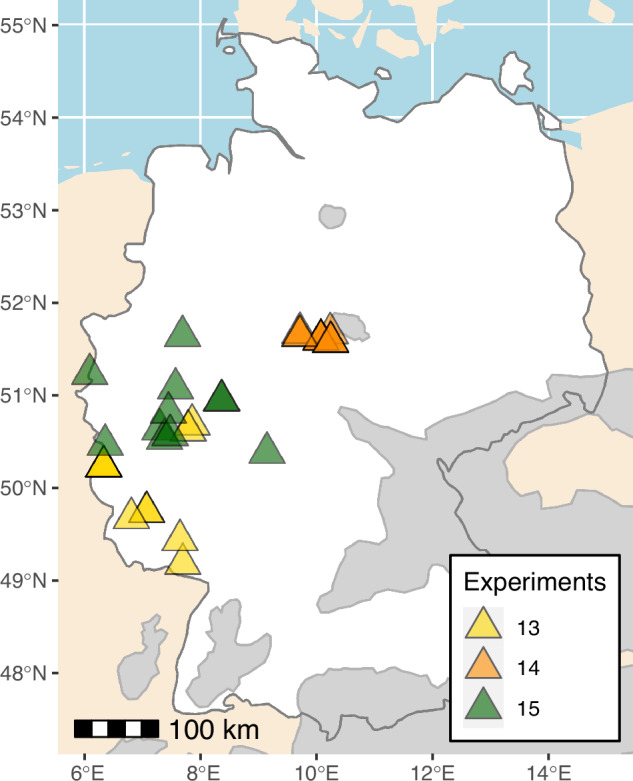
Site locations of the three German experiments. Each experiment contained 11 provenance blocks, planted at five to ten different locations. All of them were located outside of the natural distribution (grey).

Three of the 20 experiments were established in Germany, namely EXP13, EXP14 and EXP15. EXP13 and EXP14 were planted in April/May 1968 with 4-year-old nursery plants, similar to most other European experiments, while EXP15 was planted one year later with 5-year-old planting stock. Due to lack of large enough forest sites to establish 11 provenance blocks at the same spot, all three experiments were split with one to four blocks of 100 provenances planted at different locations. EXP13 consisted of seven different site locations in Rhineland-Palatinate, with a maximum of four provenance blocks located in Brandscheid. EXP14 consisted of five locations in Lower Saxony, with four blocks of highly similar conditions in Holzerode and three blocks in Rüdershausen. EXP15 was split to 10 locations in North Rhine-Westphalia, Rhineland-Palatinate and Hesse. In total, 33 sites with 20 replicates of 100 provenances (surrounded by one row of border plants) were planted in western Germany (Fig. 2).

In a few cases the origin of planted individuals was unclear (0 to 78 individuals depending on trial site). Either labels were lost during transport and planting, or if plants were missing completely, their empty planting spot was filled with a border plant of unknown origin. In consequence, the number of trees of known origin ranged from 1922 to 2000 trees/site at the time of establishment.

### Measurement of phenotypic traits

**Fig. 3:**
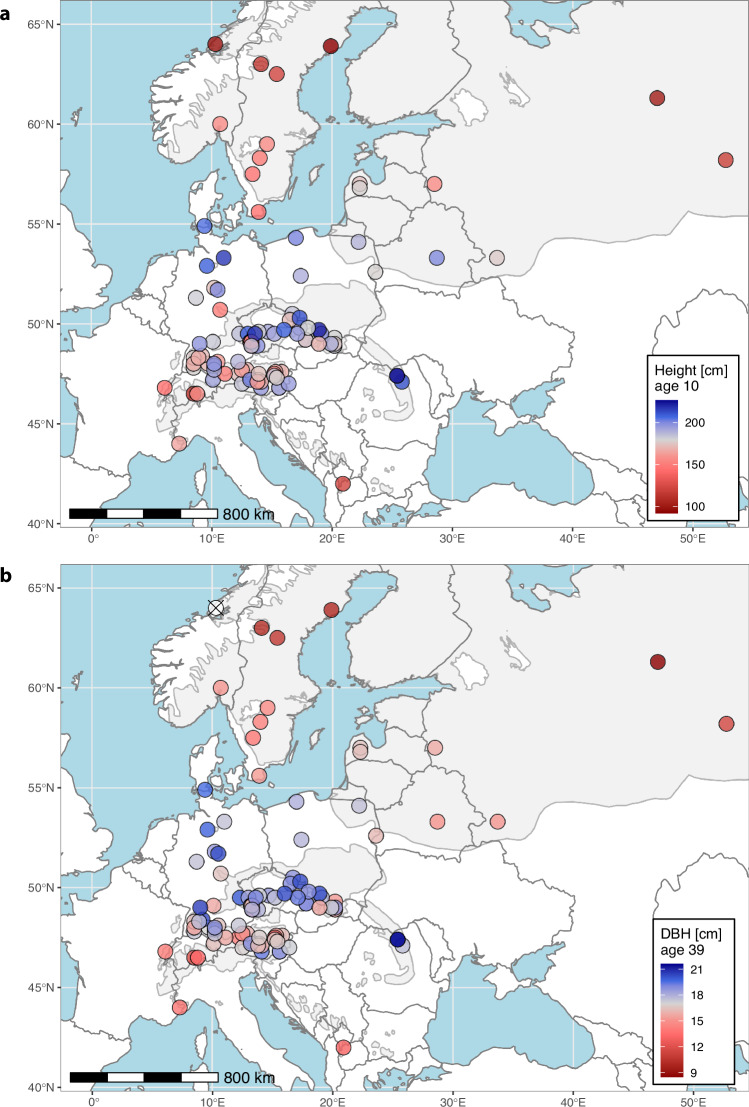
Provenance mean performance plotted at their location of origin. Exemplarily, means are given for one trial site (SITE_ID = 1301). (**a**) Tree height [cm] at age 10 and (**b**) diameter at breast height [cm] at age 39 are averaged by provenance. One provenance in Norway was completely missing at age 39 and is marked with a cross ⦻. The natural distribution of *Picea abies* in grey in the background is reproduced from EUFORGEN^16^.

All German sites were measured in parallel. Growth parameters were assessed during dormancy, with tree age being assigned according to the last concluded growing season. Height was recorded at recurring time intervals of three years in 1970 (age 7), 1973 (age 10), 1976 (age 13) and 1979 (age 16) using a measuring pole. Afterwards the focus switched to diameter at breast height (DBH) measured with a tree caliper or diameter tape at 1.3 m above ground. For DBH time intervals were larger and irregular. It was measured in 1979 (age 16), 1984 (age 21), 1992 (age 29), 2002 (age 39) and 2014 (age 51). As measurements were taken for all remaining individuals, survival can be inferred as proportion of living vs. originally planted trees (i.e. the number of rows per provenance and site). However, it becomes biased with the first occurrence of thinnings. Prior to the measurements in 1984 (age 21) there was no silvicultural thinning across all 33 sites. The management regime followed silvicultural considerations, but varied in timing and intensity among sites, as local forest administrations and/or owners were responsible. Figure 3 gives an example how this data can be plotted and further analysed by trait for individual sites.

Several sites were subjected to severe bark beetle infestations (*Ips typhographus*) since the last assessment and the series was finally declared closed after 2014.

## Data Records

The database^15^ consists of three independent comma-separated value (csv) files and one corresponding metadata descriptor document (Pabies_metadata.pdf) available in the OpenAgrar data repository (10.3220/DATA20240507093138-0). The first file Pabies_prov.csv contains geographic information of seed origin for 1,100 provenances provided after seed collection and the second Pabies_sites.csv the geographic information of 33 German trial sites. The third file Pabies_phenotypes.csv provides individual-tree based phenotypic measurements of height [cm] and diameter at breast height [cm] assessed for 65,518 trees in the field at varying time intervals between age 7 to 51 (Table 1). In total, the dataset includes 424,781 phenotypic records.

**Table 1:** Number of individual-tree-based records taken at each time interval summarized at experimental level.

Trait	Height				Diameter				
Year	1970	1973	1976	1979	1979^(1)^	1984	1992	2002	2014
Age	7	10	13	16	16	21	29	39	51
**Experiment**									
EXP 13	19,562	19,180	18,923	18,769	18,532	18,518	16,316	9,696	5,187
EXP 14	19,932	18,676	18,409	18,313	18,123	18,177	16,064	7,906	4,013
EXP 15	20,575	18,960	18,195	17,711	17,502	17,313	15,875	8,954	5,391
∑	60,069	56,816	55,527	54,793	54,157	54,008	48,255	26,556	14,591

^(1)^The number of records for DBH at age 16 are lower than those for height at same age because trees that did not yet reach breast height (<1.3 m) were not measured.

## Technical Validation

The database of phenotypic records has been validated at different stages during the entire observation period. Prior to publication of the data, it was carefully screened for consistency across measurement intervals. Plausibility of individual data records was checked by subtraction of the preceding measure from the following and correlations between measurements. Irregularities (e.g., shrinking trees), extreme values and outliers were identified and compared with the hand written field documents to clarify these deviations and correct data entry errors if possible. The consistent development of increasing growth with age but decreasing survival is shown at site level in Table 2.

**Table 2:** Summary table with site means for growth traits and proportion of survival prior to silvicultural thinning.

Trait	Height [cm]				Diameter [cm]					Survival [%]				
Year	1970	1973	1976	1979	1979	1984	1992	2002	2014^(1)^	1970	1973	1976	1979^(2)^	1984
Age	7	10	13	16	16	21	29	39	51	7	10	13	16	21
Site														
1301	63	175	321	480	6.3	10.0	13.4	17.3	23.5	95.3	94.9	94.2	93.8	93.0
1302	60	160	290	429	5.9	10.3	13.5	22.4	28.5	83.2	80.9	79.3	78.6	77.0
1303	56	161	296	438	5.8	9.7	12.8	21.4	27.3	74.4	73.2	72.5	71.6	71.0
1304	66	179	334	500	6.5	10.0	13.5	17.9	24.6	96.6	95.7	95.2	94.8	94.5
1305	60	154	280	420	5.6	9.7	13.6	21.3	30.3	94.7	92.2	90.7	89.9	88.8
1306	63	159	275	393	5.1	9.2	12.3	20.3	25.3	88.6	85.3	83.0	82.1	79.9
1307	60	160	279	372	4.0	7.0	10.6	13.9	17.0	92.0	88.2	87.2	86.3	85.7
1308	77	189	350	544	7.5	11.1	17.2	22.7	NA	96.9	96.6	94.8	94.7	92.8
1309	60	166	331	526	7.5	10.4	14.9	19.8	32.0	86.2	84.4	82.5	81.0	80.0
1310	61	156	275	405	5.2	9.2	12.1	21.0	27.0	84.3	84.4	83.6	82.7	80.6
1311	59	142	276	443	6.5	9.5	12.8	21.5	27.1	95.3	92.7	92.4	91.9	91.4
1401	54	149	310	480	5.8	9.3	12.0	18.5	26.5	92.4	91.0	90.1	90.0	88.9
1402	52	149	304	479	7.4	9.9	13.2	21.8	NA	90.1	88.9	87.5	86.2	85.5
1403	67	177	333	501	6.3	9.8	13.7	21.3	27.7	93.7	89.1	88.1	87.7	87.1
1404	69	178	342	512	6.1	9.6	13.5	18.5	24.8	97.6	94.6	93.4	92.1	91.8
1405	70	189	390	590	7.8	11.6	16.2	24.3	32.5	76.3	75.2	74.5	74.3	74.0
1406	57	153	316	499	6.0	9.8	12.9	19.3	25.9	87.3	85.9	84.3	84.1	82.7
1407	56	148	318	499	6.2	9.4	13.0	19.3	27.7	93.0	90.8	89.4	89.5	88.6
1408	60	162	331	489	5.7	8.8	12.0	18.2	24.2	92.3	91.8	90.2	90.2	89.7
1409	69	175	329	490	5.7	9.6	13.5	19.7	26.5	94.7	91.9	90.3	89.9	89.1
1410	54	149	299	461	7.2	10.2	13.0	21.2	29.8	88.3	86.2	84.8	84.4	84.1
1411	57	151	297	470	7.5	10.4	13.5	21.2	29.2	91.6	49.2	48.6	48.2	48.2
1501	45	117	274	479	7.1	11.3	15.3	25.1	33.2	99	96.5	96.1	95.7	92.9
1502	41	98	173	325	3.9	8.5	13.7	26.2	34.9	84.6	69.3	58.7	53.1	49.3
1503	46	112	223	419	5.2	9.5	14.2	20.1	24.6	95.3	87.1	84.7	79.0	75.2
1504	44	117	263	457	7.1	10.2	14.2	20.2	NA	99.0	99.0	97.8	97.6	97.6
1505	47	95	198	351	4.9	8.0	13.4	22.5	29.3	94.9	86.6	85.5	84.8	83.2
1506	48	125	237	411	6.1	9.4	12.8	20.4	29.6	97.4	91.6	84.9	80.6	79.3
1507	44	105	216	412	6.7	10.7	15.6	20.6	25.1	95.8	87.7	85.1	83.4	82.7
1508	46	101	205	398	6.1	10.1	14.5	19.6	23.5	93.9	83.8	80.3	79.5	78.3
1509	49	122	214	337	4.3	7.2	10.5	16.5	23.8	95.5	90.5	87.9	86.3	84.0
1510	45	113	242	406	5.5	10.9	14.9	23.5	36.3	87.5	75.3	69.8	68.8	68.0
1511	51	134	265	442	6.4	9.4	12.5	19.3	25.2	97.5	91.4	89.5	87.3	85.2

^(1)^Three sites left blank in 2014 were already abandoned at time of the final assessment.

^(2)^Survival in 1979 is based on the number of individuals measured for tree height.

## Usage Notes

Initially, the series was planned for an active observation of 20 years^6^, as complete assessments are only possible prior to any thinnings that inevitably cause rank changes for such experimental design with single-tree-plots^11^. Nevertheless, valuing the immense efforts taken with this trial, observations were continued at the German trial sites for a total of five decades. Measures recorded in 1992, but even more those from 2002 and 2014 have to be evaluated with caution. In 1984 82% of the initial number of trees (54,008 of initially 65,518) were still alive, representing natural dieback due to environmental conditions (Table 1). This proportion decreases with the effect of silvicultural treatments first to 73% in 1992, then to 40% in 2002 and finally to 22% in 2014. Three sites were already abandoned completely by the final assessment.

## Data Availability

Four files with R code are provided together with the data (10.3220/DATA20240507093138-0). The first R code displays provenances and sites according to their geographic location (location_of_provenances_and_sites.R) and the second merges metadata to phenotypes (merge_metadata_to_phenotypes.R). The third calculates arithmetic trait means per provenance for one individual site and displays these spatially at their corresponding geographic location (mean_growth_by_provenance.R). The fourth illustrates the experimental design and the spatial distribution of single tree-based measurements for each individual site (experimental_design_by_site.R).
